# Diet and wild ungulate preferences of wolves in northwestern Anatolia during winter

**DOI:** 10.7717/peerj.7446

**Published:** 2019-08-21

**Authors:** Deniz Mengüllüoğlu, Eylül İlaslan, Hasan Emir, Anne Berger

**Affiliations:** 1 Department of Evolutionary Ecology, Leibniz Institute for Zoo and Wildlife Research (IZW Berlin), Berlin, Germany; 2 Department of Biology, Chemistry and Pharmacy, Freie Universität Berlin, Berlin, Germany; 3 Tüylü Dostlar Veteriner Kliniği, Aydın, Turkey; 4 Wildlife Department (WDT), Turkish Ministry of Agriculture and Forestry, Ankara, Turkey

**Keywords:** *Canis lupus*, Predator-prey, Sus scrofa, Prey preferences, Livestock guarding dog, Population density, *Ovis gmelinii anatolica*, Human-wildlife conflict

## Abstract

The gray wolf (*Canis lupus*) is making a comeback in many habitats in central Europe, where it has been once extirpated. Although densities are still low to moderate, this comeback already raises management concerns. In Anatolia, the gray wolf is one of the most common predator species occupying almost all kind of habitats. Although its numbers were reduced in some parts of the country, it has never been extirpated and lived in sympatry with humans. In this study we investigated, for the first time, the winter diet of wolves in north-west Anatolia, where a multispecies wild ungulate community occurs in sympatry with high density livestock. We selected two geographically close but different habitats (steppe and forest) with different wild prey availabilities and compositions. In both areas ungulate contribution to winter diet biomass was more than 90%. Wolf pack size (four to eight wolves) were higher in the study area where livestock numbers and human disturbance were lower and wild prey were more available. In both study areas, wild boar (*Sus scrofa*) was the main and most preferred food item (Chesson’s α = 0.7 − 0.9) and it occurred at higher density where wolf pack size was smaller. We could not find a high preference (Chesson’s α = 0.3) and high winter predation pressure on the reintroduced Anatolian wild sheep (*Ovis gmelinii anatolica*) population that occurs in the study area covered by steppe vegetation. Contribution of livestock and food categories other than wild ungulates to wolf diet stayed low. Wolves can help mitigate human-wildlife conflict regulating wild boar numbers, the most common conflict-causing ungulate species in Anatolia. Instead of managing wolf numbers in human dominated landscapes, we recommend reintroduction of wild ungulates to the areas where they became locally extinct and replaced by livestock.

## Introduction

The gray wolf (*Canis lupus*) is one of the most common predator species (6,000–8,000 individuals: Ambarli, Ertürk & Soyumert, 2016) occupying Anatolia (Asian part of Turkey), Turkey. It inhabits almost all type of habitats, ranging from deciduous Black sea forests to open steppes of middle and semi-deserts of south-eastern Anatolia (Ambarli, Ertürk & Soyumert, 2016; Çoğal & Sözen, 2017). As an apex predator, the wolf plays a crucial role in the ecosystems by regulating large ungulate and meso-predator numbers (Ripple et al., 2014). Studies have shown that where wolves are extirpated, ungulate numbers might rise dramatically, causing reduction of biological and morphological diversity in terrestrial and aquatic ecosystems (Ripple et al., 2014). On the other hand, where ungulate numbers and biodiversity are dramatically reduced by anthropogenic factors (e.g., poaching, habitat destruction and alteration, excessive farming and grazing by livestock) wolves can be a major cause of human-wildlife conflict (Tuğ, 2005; Soofi et al., 2019).

The wolf is making a comeback in many habitats in central Europe, where it had once been extirpated (Chapron et al., 2014). Although densities are low to medium, this comeback raises management concerns due to long time absence of the species (Linnell & Boitani, 2012) and lost traditional husbandry methods (i.e., livestock guarding) during this time period (Chapron et al., 2014). In Anatolia, the wolf has never been extirpated and it coexisted with human (Ambarli, Ertürk & Soyumert, 2016) and livestock in most of its distribution, and traditional livestock grazing with livestock guard dogs and shepherds is still being applied throughout the wolf’s distribution (Rigg, 2001).

In central and eastern Anatolia, where animal and crop farming are the main anthropogenic activities and wild prey are scarce, wolves depredate on livestock at high rates (Tuğ, 2005; Capitani et al., 2016). However, no study in Anatolia has questioned how the wolf diet is in an area where wolves coexist with wild and domestic prey communities. Studies have shown that when wild ungulates are available, wolves prefer to prey on these prey species instead of livestock (Zlatanova et al., 2014). Therefore, our alternative hypothesis to livestock being main prey of wolves in Anatolia is, given reliable source of wild prey, that wolves prefer wild ungulates, and that livestock contribution to wolf diet remains low even in high density livestock presence.

**Figure 1:**
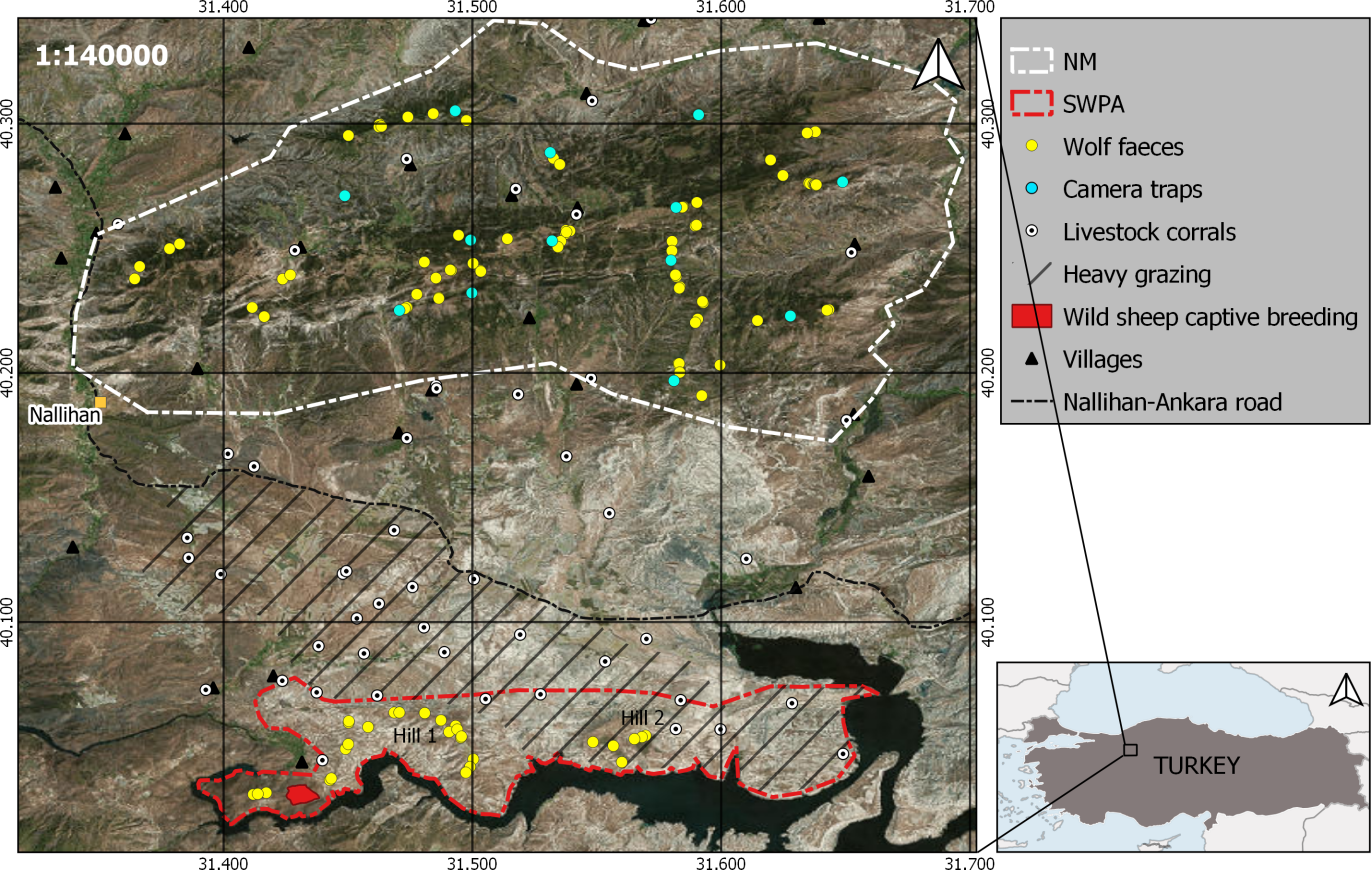
Locations and boundaries of the two study areas in Turkey. Map showing vegetation cover, locations of collected wolf faeces (yellow dots) and camera traps (blue dots) in NM, livestock heavy grazing area in and around SWPA (line fill), Anatolian wild sheep captive breeding centre (red polygon) in SWPA, livestock corrals (white dots) and villages (triangles) around two study areas. 10 km to 10 km grid is set to visualize distance between the two study areas. This figure has been produced using freely available SRTM Worldwide elevation and World Imagery (https://earthexplorer.usgs.gov/) and shape files (http://www.naturalearthdata.com).

In some protected areas, wolves are seen as the main cause of losses in reintroduced ungulate populations by the Wildlife Department of Turkey (WDT). Reintroduced Anatolian wild sheep (*Ovis gmelinii anatolica*) in the Sarıyar Wildlife Protection Area (SWPA; Fig. 1), northwestern Turkey, is such a case in point (Özüt, 2009). In spite of the large amount of money investment and several re-stockings of wild sheep, its numbers haven’t increased and have been oscillating around 40–80 sheep in the period between 2005 and 2017. The habitat preferences of the Anatolian wild sheep do not allow it to disperse to other nearby habitats in the west and north (mountains with high vegetation cover) and it therefore only occupies open hilly habitats in SWPA (Özüt, 2009). The old migration route of wild sheep towards southeast (hills and mountains covered with steppe ecosystem; Turan, 1984) here has been cut by the construction of Sarıyar Dam in 1956, which was the reason for wild sheep local extinction in the area. Therefore, wild sheep in SWPA are only confined to two main hills in the center of the protection area (Fig. 1; Hill 1 and 2); they cannot display seasonal area shifts and are continuously influenced by anthropogenic factors such as livestock grazing and LGD. Since 2005, there have been several attempts from the WDT to manage the predator numbers such as wolves and jackals (*Canis aureus*) in the area; however, these attempts did not reveal any success in wild sheep growth rates (WDT, 2005–2017, unpublished data). Despite the argument that wolves may be the reason for stagnant sheep population development, there has never been any study focusing on the wolf-prey dynamics in SWPA and the real influence of the local wolf population on wild sheep. Therefore, we also aimed to quantify the impact of the wolf predation on Anatolian wild sheep in SWPA during winter.

To test our hypothesis, we conducted this study to reveal the diet and wild ungulate preferences of wolves in north-western Anatolia. We selected two closely located study areas with different habitat types and different composition of multi-species wild and domestic ungulate communities. Here, we quantified wild ungulate densities and availabilities using annual inventories’ and camera trapping data and consumed prey biomass through faecal diet analysis. Finally, we compared diets, prey preferences, niche widths and niche overlap of wolves in our two study areas during winter.

## Materials and Methods

### Study areas

Our study areas are both located inside the borders of Nallıhan district of Ankara province, where the mean human population density is 14 individuals/km^2^.

The SWPA hills (80 km^2^) are mostly covered with dry steppe vegetation (Fig. 1), where scattered oak (*Quercus pubescens*) and juniper scrub (*Juniperus excelsa* and *J. oxycedrus*) occur on the slopes. The elevation of the SWPA varies between 460 m and 850 m above sea level (asl). The area is surrounded by the Sarıyar Dam in the east, south and west, and by grazing pastures and agricultural fields in the north (Fig. 1). The climate around SWPA is warm and temperate (annual mean temp. = 13.1 °C). Most of the precipitation fall as rain and snow cover stays for a mean number of seven days (maximum depth of 25 cm) (Turkish State Meterological Service, 2012, unpublished data; CLIMATE-DATA.ORG, 2019a). The human population around the SWPA is very low and occurs in three settlements in the northwest of SWPA. Half of the registered human population (N_total_ = 926; (TURKSTAT, 2017) reside in Nallıhan district center or close by big cities (D Mengüllüoğlu, pers. comm., 2014–2016, with village heads). In flat lowlands, in the south of Nallıhan-Ankara road (Fig. 1), agricultural fields and livestock grazing pastures occur with very high livestock (53.8 sheep and goats/km^2^; 2016 inventory of Nallıhan Directorate of Agriculture) and livestock corral density (0.16 corrals/km^2^). They graze freely during day time and continuously guarded by livestock guarding dogs (LGD; depending on the flock size 2 to 6 dogs per flock) and shepherds (generally one shepherd per flock). As in other parts of Anatolia, flocks are kept inside corrals (where also the shepherd/s live) surrounded by pens and protected by unleashed LGD during night time (Tuğ, 2005). While grazing in the western half of SWPA is strictly prohibited, it is allowed and very common in the eastern half (Fig. 1). However, flocks are regularly invading the northern slopes and hill tops of the strictly protected area. Every summer, some of the larger livestock flocks move to the further north of the Nallıhan Mountains (Fig. 1) for the more productive and cooler highlands. During winter and spring, however, due to the calmer climatic conditions, all flocks are grazed around SWPA and all livestock corrals (*n* = 29) are active. Potential wolf wild prey species in SWPA are red deer (*Cervus elaphus*), wild boar (*Sus scrofa*), reintroduced Anatolian wild sheep and brown hare (*Lepus europaeus*) (Özüt, 2009). Due to the intensive grazing activity in the northern part, wild ungulates mostly occur in the southern slopes of SWPA facing the Sarıyar Dam Lake (authors’ and WDT staff’s observations, 2005–2017; Fig. 1).

About 15 km north of the SWPA, the second study area the Nallıhan Mountains (NM; 400 km^2^ in size) is covered by Turkish pine (*Pinus brutia*) at lower elevations (500 to 1,000 m asl) and black pine (*Pinus nigra*) and junipers with an understory of oak-dominated scrub (*Pyrus elaeagnifolia*, *Crataegus spp*.; Aksoy, 2009) at higher elevations (800 to 1,500 m asl). The mean annual temperature is 9.6 °C and the mean annual total precipitation is 543 mm (CLIMATE-DATA.ORG, 2019b). Due to the higher elevational gradient in NM, snow stays much longer than in SWPA and during winters 2014–2015 and 2016–2017 snow cover stayed more than one month above 1,000 m asl. The human population in and around NM is very low (12 villages, N_total_ = 1780; (TURKSTAT, 2017) and more than half of the registered population (mostly young people) resides in Nallıhan district center or close by big cities such as Ankara, Eskişehir and Bolu (D Mengüllüoğlu, pers. comm., 2014–2016, with village heads). Contrary to the SWPA, no livestock flocks were being grazed at high elevations in NM during winter and early spring time, when we collected faecal samples. Livestock stayed close to villages in the valleys and flatland at lower elevations. Majority of the livestock corrals here are inside the villages (Fig. 1). Wild prey species in NM are red deer, wild boar and brown hare (Mengüllüoğlu et al., 2018).

The region covering both study areas is home to several other large- and meso-carnivores, such as brown bear (*Ursus arctos)*, Eurasian lynx (*Lynx lynx*), golden jackal (*Canis aureus*), red fox (*Vulpes vulpes*) and jungle cat (*Felis chaus*) (Mengüllüoğlu, 2010; Mengüllüoğlu et al., 2018). Brown bear and lynx, however, occur only sporadically in the SWPA (Özüt, 2009; D Mengüllüoğlu, pers. obs., 2017).

### Faeces collection and diet analysis

Wolf faeces were collected between 2014 and 2017 during winter and early spring seasons (December–April; Wildlife Department of Turkish Ministry of Agriculture and Forestry permit number: 30057506-030-1867). At seven survey days during winters 2015–2016 and 2016–2017 (December–February) we covered the wildlife trails and dirt roads on three main hill tops of the SWPA (total effort: 71.2 km) and collected 28 wolf faeces. Eighty-one wolf faeces at NM were collected mainly at wolf territorial marking sites and when encountered on dirt roads during Eurasian lynx (*Lynx lynx dinniki*) daily live trap control trips (December-early April: 2014–2015, 2015–2016, 2016–2017 trapping seasons; (Mengüllüoğlu et al., 2019). Due to the regular wolf marking site controls and continuous sample collection we did not collect samples originating from summer or autumn seasons and majority of faeces were at most one week old. Our surveys did not include the reproduction periods of wild sheep and red deer in SWPA and NM, however partially overlapped with early wild boar reproduction period only in NM. To avoid misidentification and possible confusion with livestock guarding and hunting dogs’ faeces, which occur more in the SWPA, only faeces with wild and domestic prey remains (hair and bones) were collected applying SCALPS criteria (i.e., depending on size and shape and wolf marking signs; Reinhardt et al., 2015). Several faeces (*n* = 4) with human sourced food content (bones together with plastic remains) were removed from the analysis to prevent designation of dog faeces as wolves’.

Faeces were oven-dried and washed following the protocols of Wagner et al. (2012). Prey remains such as hair, bones, teeth, nails and feathers were separated and weighed. Hairs were classified according to their microstructure and identified with the help of a reference book (Teerink, 1991) or by comparing them with local wildlife and livestock reference collections taken from the Berlin Natural History Museum and shepherds (see Supplemental Information). After classification, the frequency of occurrence (FO) of each species in the diet was noted. For the purpose of estimating the consumed biomass per prey species, we used the correction factors (CF) of wolf regression model of Wachter et al. (2012), which was applied to the results of European and Indian wolf feeding experiments conducted by Rühe, Buschmann & Wameling (2003) and Jethva & Jhala (2004), respectively. We calculated the consumed mass of each prey species per faeces and then multiplied this value with the total ingested volumes. For species such as domestic livestock and rodents that were not included in Rühe, Buschmann & Wameling’s (2003) experiment, we used the CF of Jethva & Jhala (2004) in Wachter et al. (2012). However, for prey species that were not included in both experiments we directly applied European wolf exponential regression model of Wachter et al. (2012) on average body weight of prey species and obtained consumed biomass per faeces.

### Wild ungulate densities and wolf pack size

Herbivore prey abundances in the SWPA were provided from 2016 late autumn (November 24th, 2016) inventory count by the WDT. Every year, two total wildlife count inventories, one in summer after ungulate reproduction period and one in late autumn during wild sheep rut period (November–December) are conducted by 15 to 20 WDT staff. Counts are performed simultaneously at ten vantage points distributed over SWPA hill tops where observers count the human driven wildlife from lower elevations towards the hill tops. As wild boars and red deer are also observed around the croplands close to settlements in the noth-west of SWPA and have distributions in farther west, we extrapolated densities of these two species for 100 km^2^ in estimating available biomasses. However, due to its habitat preferences and gathering during rut, unlike red deer and wild boar wild sheep is only confined to SWPA hills. Therefore, we did not extrapolate the wild sheep numbers but instead used total WDT count for density per 100 km^2^. The wolf pack size in the SWPA was estimated from inventory counts, field observations and based on opportunistic camera trapping of WDT in the SWPA in previous years and during our survey periods.

As driving counts were not possible in NM due to large size and much denser vegetation cover, we used the Random Encounter Model (REM; (Rowcliffe et al., 2008) to estimate wild ungulate densities. We estimated the species’ densities (D) by $D= \frac{y}{t} \ast \frac{\pi }{Vr \left( 2+\theta \right) } $, where *y* is the number of independent photographic events, *t* is camera trap days (ctd), *V* is average speed of animal movement, *r* and *θ* are the camera trap detection distance (in kilometer) and angle (in radian) respectively. Movement speeds of animals from similar habitats (southern Europe) were taken from published literature with GPS fix frequencies of 15 min for red deer (Pépin et al., 2004) and wild boar (Spitz & Janeau, 1990). In the model, we used data from our camera traps that have been running all year round since 2014 for the purpose of lynx population monitoring in NM (Mengüllüoğlu et al., 2019). To meet closed population assumption we restricted camera trapping duration to December–February in winter 2016–2017 which made up 1,080 camera trap days by thirteen camera trap stations (Fig. 1). Since most of the ungulate seasonal migrations occur in late autumn-early winter (continuous camera trapping since 2014), we assumed the ungulate population was stable during winter. Wolf packs were also present throughout the survey period (proofed by faeces, tracks, and camera trapping data). The camera trap stations were installed different elevations and kind of habitats at a mean nearest neighbour distance of 3.5 ± 0.9 km to maximize the lynx captures and therefore were randomly placed with respect to red deer and wild boar movements (please see Supplementary Information 2 for more detailed information on stations). One trap station was installed at a lynx marking point, however, as the lynx in the study area are lagomorph specialists (Mengüllüoğlu et al., 2018), we assumed ungulate movements during winter were not influenced by lynx movements. The stations were covering a minimum convex polygon of 148 km^2^ and distributed throughout NM excluding the western part (camera traps here were removed during summer 2016 due to the active logging by forestry department). Cameras were set to record one minute videos to estimate the average group sizes of red deer and wild boar in order to obtain a reliable REM density. We set a minimum interval of 30 min to assign two videos of the same species as independent captures. Delta method (Seber, 1982) was used to calculate 95% of confidence intervals for the estimated densities.

From inventory counts (the SWPA) and from REM densities (NM), mean prey biomass per kilometer square was calculated by using mean winter body weights of 150 kg for red deer, 40 kg for wild sheep and 120 kg for wild boar (Turan, 1984). We did not calculate available biomass for domestic livestock as this food category was continuously guarded by shepherds and LGD, and was not directly available for wolves.

### Prey preferences, niche overlap and width

Chesson’s selectivity index, α (Chesson, 1978) was applied on wild ungulate prey to assess wolf prey preferences. We did not quantify domestic prey selectivity. In order to quantify the trophic niche overlap between the wolf packs in SWPA and NM, we applied Pianka’s overlap index (Pianka, 1973) on five prey categories, Anatolian wild sheep, red deer, wild boar, domestic livestock and others. To quantify and compare the niche width of the wolves in SWPA and NM, we applied standardized Levin’s index (Hurlbert, 1978) on the five prey categories mentioned above.

## Results

### Diet

**Figure 2:**
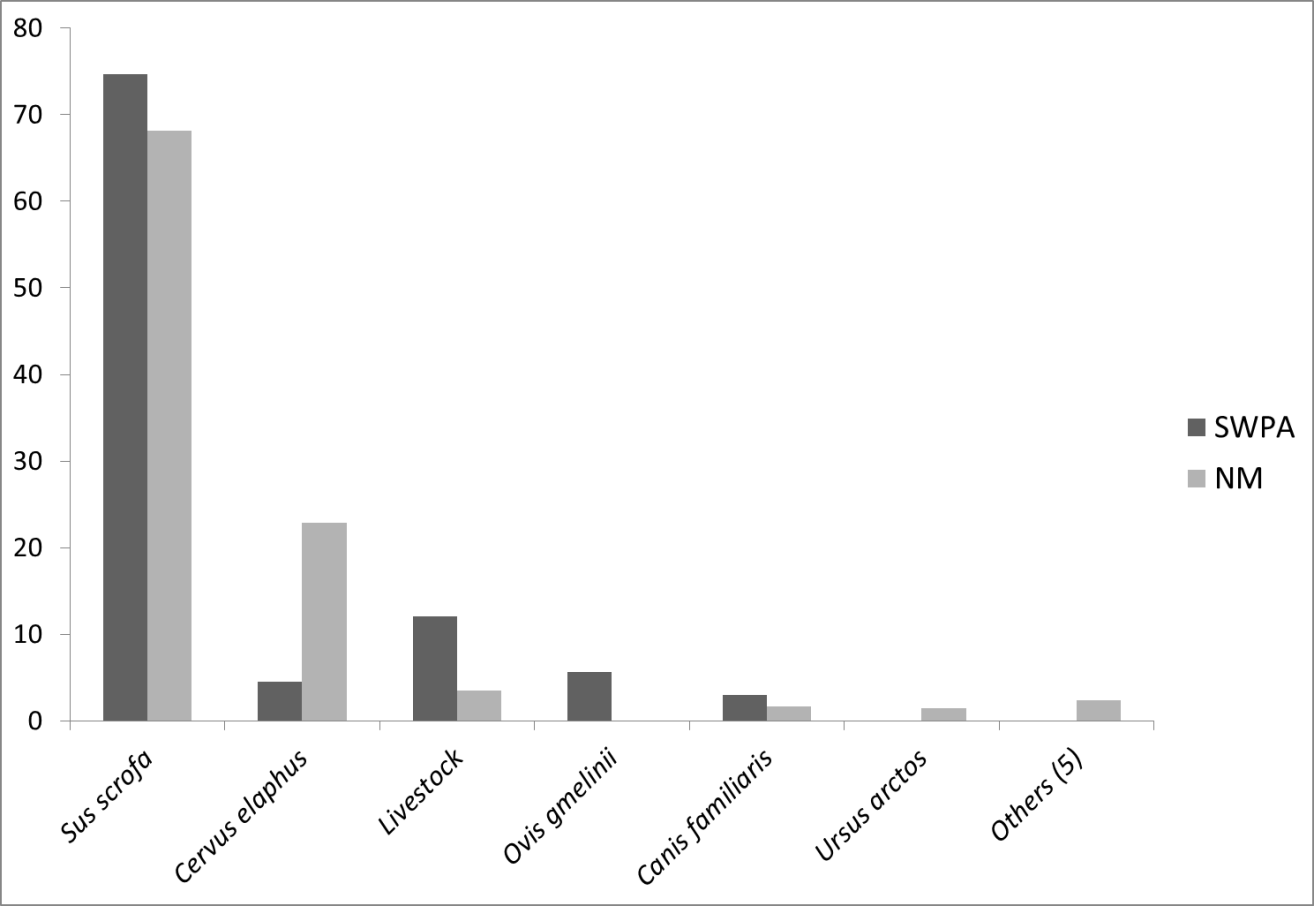
Percentages of consumed prey biomass in wolf diet in the SWPA and NM. Others category includes brown hare, Eurasian badger, rodents, poultry and plant material encountered in wolf diet only in NM.

Faecal analyses revealed five and ten prey items for the SWPA and NM, respectively (Table 1). Wild ungulates formed 85% of the diet biomass in the SWPA and 91% in NM. Wild boar was the main prey species and its contribution was 75% in the SWPA and 68% in NM (Fig. 2). However, ungulate species with the second highest biomass contribution in winter diet differed between the two areas, being wild sheep in the SWPA and red deer in NM (6% and 23% respectively). Winter diet of wolves in SWPA did not indicate high predation on Anatolian wild sheep. Livestock had a higher contribution to wolf diet in the SWPA (12%) compared to livestock in NM (3.5%). Domestic dog was a common prey, however its percentage did not exceed 3% in neither of the survey areas (Fig. 2). In NM, brown bear, Eurasian badger (*Meles meles*), rodents and poultry have also contributed to wolf diet in very low rates (Table 1).

### Wild ungulate availability and wolf pack size

Late autumn inventory in 2016 revealed 58 wild sheep, 53 red deer and 88 wild boars in the SWPA (80 km^2^). The densities of ungulates were therefore 58 wild sheep, 66.3 red deer and 110 wild boars per 100 km^2^ (Table 2). The wolf encounters (*n* = 3) during our faeces collection surveys, annual inventories of WDT (*n* = 2) and camera trapping records of WDT (*n* = 8) indicated that the wolf pack in the SWPA (80 km^2^) is composed of two adult and one to two sub-adult wolves per year.

In NM, camera traps encountered two ungulate species with 73 events for red deer and 24 events for wild boar. Average group sizes from camera trap videos were estimated to be 1.8 and 2.9 individuals for red deer and wild boar, respectively. We estimated wolf group size per capture in NM from camera trap videos (*n* = 7) as 3.4 individuals (one to eight fully grown adults and sub-adults together). The pack sizes however, were determined to be a minimum of four to eight wolves per four encountered packs. REM resulted in densities of 227 red deer/100 km^2^ and 83 wild boar/100 km^2^ (Table 3).

**Table 1:** Wolf diet in two study areas (SWPA and NM) in north-west Anatolia, expressed as frequency of occurrences (FO), relative frequency of occurrences (%FO), relative volume (% Vol) and relative biomass (% Bio).

		**SWPA** (*n* = 28)				**NM** (*n* = 81)			
Species	Kg consumed per faeces	FO	% FO	% Vol	% Bio	FO	% FO	% Vol	% Bio
**Wild prey**									
*Ovis gmelinii*	0.25	3	10.34	10.71	5.69				
*Cervus elaphus*	0.43	2	6.89	5	4.56	20	22.73	23.1	22.84
*Sus scrofa*	0.53/0.18^a^	19	65.51	66.36	74.66	50	56.82	58.33	68.15
*Ursus arctos*	0.52					1	1.14	1.23	1.47
*Meles meles*	0.15					2	2.27	2.47	0.85
*Lepus europaeus*	0.12					3	3.41	3.7	1.02
*Microtus sp.*	0.09					1	1.14	1.23	0.28
**Domestic prey**									
*Canis familiaris*	0.2	2	6.89	7.14	3.03	3	3.41	3.7	1.7
*Capra hircus*	0.25					1	1.14	1.23	1.96
*Ovis aries*	0.53	3	10.34	10.71	12.06	1	1.14	1.23	1.51
*Gallus gallus*	0.15					1	1.14	0.62	0.21
**Plant material**	^b^					5	5.68	3.15	^b^

**Notes.**

a for piglets.

b not applicable.

**Table 2:** The 2016 late autumn inventory counts (total number) and densities of wild ungulates in the SWPA (WDT 2016 Inventory).

**Species**	**Inventory count (80 km^−2^)**	**Density (100 km^−2^)**
Red deer	53	66.3
Wild sheep	58	58
Wild boar	88	110

**Table 3:** Parameters used in the Random Encounter Model (REM), winter group sizes and winter densities of red deer and wild boar in the study area Nallıhan Mountains (NM).

**Species**	**Number of captures (*y*)**	**Number of trap days (*t*)**	**Travel speed (*v; km*)**	**Radius (*r; km*)**	**Angle (*θ; radians*)**	**REM density (100 km^−2^) [lower and upper limits]**	**Average group size**	**Density (100 km^−2^)**
Red deer	73^a^		4.0 ± 1.8^b^			130 [87–183]	1.75^a^	227.5
		1080^a^		0.011^d^	0.73^d^			
Wild boar	24^a^		6.6 ± 3.2^c^			25.8 [12–39]	2.91^a^	82.9

**Notes.**

a This study.

b Pépin et al. (2004).

c Spitz & Janeau (1990).

d Meek, Ballard & Fleming (2012).

After multiplying the densities with average body masses of each ungulate species in the SWPA, the available biomass proportions revealed to be 9%, 51% and 40% for wild sheep, red deer and wild boar respectively (Table 4). The available ungulate biomass proportions in NM revealed to be 79% for red deer and 21% for wild boar. The wild ungulate prey availability in the SWPA (324 kg/km^2^) was three fourth of availability in NM (431 kg/km^2^; Table 4).

**Table 4:** Herbivore prey biomass in wolf diet and in the wild and wolf food selectivity in two study areas.

	Prey species	SWPA	NM
Biomass in diet (%)	Wild boar	74.7	68.2
	Red deer	4.6	22.8
	Wild sheep	5.7	n.p.
Biomass in wild (kg/km2)	Wild boar	130.5	90.8
	Red deer	165.0	341.3
	Wild sheep	29.0	n.p.
Biomass in wild (%)	Wild boar	40.2	21.0
	Red deer	50.9	79.0
	Wild sheep	8.9	n.a.
Chesson’s α	Wild boar	**0.72**	**0.92**
	Red deer	0.03	0.08
	Wild sheep	0.25	n.a.

**Notes.**

n.p. not present n.a. not applicable

Numbers in bold indicate high preference.

### Prey preferences, niche overlap and width

Based on the calculated ungulate proportions in wolf diets and their particular availability (kg/km^2^), Chesson selectivity index revealed wild boar to be the most preferred wolf prey in the both survey areas (α_SWPA_ = 0.7, α_NM_ = 0.9). Red deer was avoided in both areas (α_SWPA_ = 0.0, α_NM_ = 0.1). Although, α_wildsheep_ was higher than α_reddeer_ in the SWPA (α_wildsheep_ = 0.3), wild sheep was not a preferred prey species (Table 4).

Winter dietary niches of the wolf populations in the two areas almost completely overlapped (Pianka’s PI = 0.96) although wild prey availability and composition in these areas differed. Wild boar contributed to the winter wolf diet at very similar biomass percentages in both study areas. Wolves complemented the rest of the diet with non-preferred wild ungulates and domestic species. A higher wild ungulate species number did not result in the observation of a broader niche width of wolves in SWPA (Table 5; SLI_SWPA_ = 0.18, SLI_NM_ = 0.31) which is probably due to low densities and availabilities of red deer and wild sheep here. Therefore, although it was more avoided than any wild ungulate species in SWPA, domestic livestock was the second important food category (12.1% of consumed biomass) here.

**Table 5:** Levin’s (LI) and standardized Levin’s (SLI) niche width indices and Pianka’s niche overlap index (PI) for SWPA and NM.

	**SWPA**	**NM**
LI	1.73	1.9
SLI	0.18	0.3
PI	0.96	

## Discussion

In this study we present for the first time the wild ungulate prey preferences and the pack sizes of wolves from a region in Anatolia, where a multispecies wild ungulate community was present. We selected two geographically close but different habitats with different wild prey availability and compositions. Although wild ungulate availability was different in two areas, wolves had similar diets and preference patterns. Contribution of livestock and food categories other than wild ungulates was relatively low. Wild boar was the main and most preferred dietary item of wolves in our two study areas and it occurred in higher densities where wolf density was low. We did not find a strong winter predation pattern of wolves on the reintroduced Anatolian wild sheep population as previously stated by the WDT officials.

### Influence of sample size on wolf prey preferences

Sample size is an important factor in reliably assessing dietary patterns and dietary preferences of carnivore species. Jethva & Jhala (2004) mentioned that cumulative percentage of occurrences of food items was stabilized between 30 to 100 faecal samples while assessing seasonal wolf diet. Gable, Windels & Bruggink (2017) have also suggested that 10 to 20 faeces per pack/area per month were enough to more accurately assess wolf diet. Marucco, Pletscher & Boitani (2008) suggested that while comparing diets of different packs or in different areas, researchers should take number of wolf packs and wolves into account. In our study, for the single wolf pack that occupied SWPA and several packs in NM, none of the sympatric ungulate species in question were rare. Wild ungulates were regularly encountered in both study areas, during our sample collection surveys, in camera trap pictures and in inventories of WDT. Our samples size in NM was appropriate to assess winter diet of wolves; however, samples size in SWPA was around the lower required thresholds mentioned (Jethva & Jhala, 2004; Gable, Windels & Bruggink, 2017). While we had much lower samples size from SWPA, this study area was 1/5th of NM in size and was roamed by only one wolf pack composed of a maximum number of three to four wolves per year. Our data, especially from SWPA, cannot reflect the wolf predation patterns on smaller prey species such as hares, mesocarnivores and rodents. However, even with the larger sample size in NM, percentage biomass of each of these smaller prey items didn’t exceed 2% (Table 1). Considering that we tried to assess winter dietary preferences of wolves focusing on relatively common ungulate prey sources per pack (other categories were pooled together for dietary niche overlap), our analyses should substantially reflect ungulate prey preferences of wolves in our study areas.

### Wild ungulate density and biomass and wolf pack size

If we would expect combined wild and domestic ungulate biomass to directly influence wolf densities and pack size, we should have observed higher wolf densities in the SWPA. Although favorite wolf food item (wild boar) was relatively more abundant and available in SWPA we couldn’t see its positive impact on wolf numbers here. Wolf pack size in the SWPA stayed less than half of the density in NM and pack size (two to three wolves) was one third to half of the pack sizes in NM (four to eight wolves). The estimated wolf pack sizes in NM were high and comparable to those in similar habitats in southern Europe (Apollonio et al., 2004; Molnar et al., 2015; Mancinelli, Boitani & Ciucci, 2018; Mattioli et al., 2018). Based on our continuous camera trap monitoring between 2014 and 2017 in NM, wolf pack size ranged between four and nine (sub-adults and adults) (D Mengüllüoğlu, 2014–2017, unpublished data), confirming longer term higher wolf densities and larger pack sizes here. On the contrary, wild boar density in NM was lower than in the SWPA, which might indicate better density or spatial distribution regulation on this prey species (i.e., high rate of predation or boars descending to valleys and close to human settlements to avoid predation) by higher density of wolves in NM.

Although livestock might be in very high densities in and around SWPA, they are not directly available for wolves due to traditional livestock grazing methods, such as companion of livestock guarding dogs (LGD) and shepherds that actively guard the flocks during day and inside the corrals during night. In fact, shepherds have stated that wolf attacks are almost absent during day time and at livestock corrals at night, and they occur when some sheep and goats are left behind in rugged landscape in SWPA or dense vegetation cover in NM unnoticed by the shepherds (D Mengüllüoğlu, pers. comm., 2014–2017, with shepherds in SWPA and NM). Our continuous camera trapping in NM has also revealed one incident of separated Angora goat (one goat individual was visible in the captures) which was depredated at the same night and consumed in next several days by wolves (Supplementary information 2).

Presence of and encounters with LGD can influence the juvenile and sub-adult wolf survival rates, hence, the densities. The efficiency of LGD in these flocks depends on the temperament of LGD when encountering wolves but independent from LGD numbers (Tuğ, 2005). During encounters near flocks or wolf dens, wolves are commonly chased away by LGD and, if caught, vulnerable individuals such as pups, juveniles and sub-adults might be injured or killed directly (Yılmaz, 2007). Although wolf hunting is prohibited in Turkey, in the case of an attack to livestock flocks, killing the wolf is considered as self-defense by the WDT officials and generally not fined. Lastly, although we don’t know the total number of wolves shot in this area there were years when predator control was applied. These suggest that the smaller pack size in the SWPA can be attributed to higher human disturbance and wolf mortality rates. In NM, however, wolves experience fewer disturbances by flocks, LGD and humans and there is no wolf management by WDT, therefore they might occur in larger packs.

### Diet, prey preferences, niche overlap and width

Our results were in contrast to foraging ecology of wolves in central Europe, where deer species were favored in wolf diet (Jedrzejewski et al., 2012; Wagner et al., 2012). In Germany, although wild boar density was highest among available ungulates, it was the third order prey item in the consumed prey biomass (Wagner et al., 2012). We found similar dietary patterns to diets of wolves in Central Italy (Capitani et al., 2004; Mattioli et al., 2011) and wild boar was the main food item of wolves in our two study areas independent of its density. Although red deer had the highest density in NM and highest available biomass in both study areas, it was avoided as a food source and wild boar was the preferred prey. The reason of much lower contribution of red deer to wolf diet in SWPA during winter might be smaller pack size here, as killing yearling and grown deer might need higher number of wolves when compared to yearlings of wild boars (MacNulty et al., 2012). However, our data allow us make statements only on wolf dietary preferences during winter time. Preference towards deer in both study areas might be higher after calving period during summer.

Being the most common and high density ungulate species in Anatolia (Turan, 1984), wild boar represents the main food source for wolves in most natural habitats (faeces based observations of D Mengüllüoğlu, 2005–2017). Here, it is mostly hunted for population control to reduce damage on agricultural lands and there is no human consumption of it due to religious reason. Carcasses are either collected by hunters as hunting dog food or left in the field and scavenged by wild and domestic carnivores (D Mengüllüoğlu, pers. obs., 2007–2017). On the other hand, other ungulate species in the region have experienced a sharp decline in the last two centuries due to uncontrolled hunting and are still under stress of poaching depending on the locality (Şekercioğlu et al., 2011). Although ungulate populations in particular areas (like our study areas) are recovering (Mengüllüoğlu, 2010), they are far from being common along the whole wolf distribution in Anatolia. We do not have data on the dietary preferences of wolves in other areas of Turkey, yet we can state that population decrease in many autochthonous ungulate populations and increase in wild boar population sizes might be the reason of a wild boar centered dietary adjustment among wolf populations here.

In contrast to many areas in Europe (Odden, Linnell & Andersen, 2006; Imbert et al., 2016), in Anatolia, livestock flocks are very often guarded with LGD such as Kangal and Akbaş dogs (Tuğ, 2005) and more importantly every flock is leaded by one or more shepherds (generally armed in areas where wolves exist) that stay around the flocks throughout day and night. We therefore deduce that, where wild ungulates are available, wolves might prefer to prey on them, instead of risking injuries and death that might result from encounters with LGD and shepherds. If livestock would have been left without protection in its current grazing grounds in SWPA and NM, we would most probably observe much higher contribution of livestock to wolf diet. Foraging patterns of wolves where wild ungulates are scarce in Anatolia also support this hypothesis. In those areas, wolves mainly depredate on livestock, feed from waste disposal areas (Tuğ, 2005; Capitani et al., 2016) and, if the weather conditions are too harsh, LGD, stray or domestic dogs are taken from human settlements (Hürriyet, 2017a; Hürriyet, 2017b; Hürriyet, 2017c; Hürriyet, 2017d; Milliyet, 2018). Therefore, the low contribution of livestock to wolf diet in our study, might be a result of a combined human, LGD and wild ungulate mediated avoidance and should further be investigated by higher amount of dietary samples and assessment of depredation rates by interviewing shepherds with respect to protection method used (i.e., LGD, shepherd and corrals).

Some livestock contribution to diet in SWPA might be also a result of wolf scavenging on livestock carcasses left by shepherds. Livestock carcasses are generally left inside dry water irrigation channels around the grazing pastures and scavenged by raptors (i.e., cinereous vulture, *Aegypius monachus*) and domestic and wild canids (foxes and jackals; D Mengüllüoğlu, pers. obs., 2013–2017). In case of mass livestock deaths due to epidemics all carcasses are buried in deep holes and not available for scavenging.

### Role of wolves on Anatolian wild sheep mortality in SWPA and other factors

As our results showed, the winter diet of wolf in the SWPA did not indicate a high Anatolian wild sheep contribution. However, dietary patterns of wolf in summer season (when lambs are small) may indicate a different pattern and should also be investigated. Other studies showed that, the reintroduced wild sheep population in SWPA did not display a lower genetic diversity than the main population in central Anatolia (Kayim, 2008). However, in the early years of reintroduction period there has been high mortality due to paratuberculosis (2004–2008; Özüt, 2009). In the captive wild sheep population in SWPA (Fig. 1), the high rate of lamb loss due to tick borne blood toxicosis and paralysis was determined as the main factor hindering population growth (Orkun, Emir & Karaer, 2016). The high density of ticks in and around SWPA might be a result of long term livestock grazing. Although grazing was forbidden in a small area in the last decade (Fig. 1 eastern half of SWPA), flocks were invading this area illegally and therefore parasite numbers might have stayed high. Orkun, Emir & Karaer (2016) have collected 1,100 tick nymphs from only five brown hares which entered the wild sheep captive breeding area from the wild. Therefore, the high rate of lamb mortality in the captive population due to ecto-parasites might also be taking place in the wild population and should also be investigated together with wolf diet analyses during summer. Weak lambs can be easy targets for predators such as wolves, LGD and other dogs, golden jackals, foxes and raptors.

Wild sheep in SWPA cannot take the old migration routes and display seasonal shifts to other areas in southeast (Turan, 1984) due to Sarıyar Dam. Hence, it cannot spatially avoid anthropogenic disturbance, high amount of parasites, and predation pressure by wild and domestic canids. Chronic stress that might result from the factors mentioned above can lower breeding success in female wild sheep and survival of lambs (Peckarsky et al., 2008). Collectively, all of these factors might in turn cause a stable or decreasing population size of wild sheep in SWPA.

## Conclusions

In two different habitats with different wild prey compositions in north-western Anatolia, the main and preferred food item of wolves was wild boar. Every year, besides the quota hunts, country-wide driving hunts are being organized to reduce wild boar numbers. However, wolves provide free and natural wild boar population regulation, and can reduce hunting and crop-farming damage costs. We could not find evidence of high depredation by wolves on reintroduced Anatolian wild sheep during winter. However, summer diet should also be investigated to quantify the impact on wild sheep and lambs during this time of the year. Our results indicated that when wild prey (i.e., wild boar) is available and prevention measures are sustained (continuous presence of shepherds and LGD with livestock) livestock contribution to wolf diet was very low.

Therefore, we recommend wild ungulate reintroductions to suitable areas (with lower anthropogenic disturbance and livestock densities) where local ungulate populations went extinct and replaced by livestock (e.g., eastern Anatolia and more suitable habitats in western and central Anatolia). This way, the human-wildlife conflict caused by wolves and wild ungulates would be minimized and a country-wide higher biological diversity would be maintained in coexistence with humans.

##  Supplemental Information

10.7717/peerj.7446/supp-1 Supplemental Information 1 Camera trap picture series in NM showing wolf depredation on Angora goat/s separated from the flock and left behind unnoticed by the shepherd Probably more than one goat was depredated as the wolf pack continued to visit the spot and carry goat parts following several days after depredation. Flock owners have also visited the spot several times searching for separated goat/s.

10.7717/peerj.7446/supp-2 Supplemental Information 2 Wolf dietary content (volume%) and prey biomass calculations

10.7717/peerj.7446/supp-3 Supplemental Information 3 Information on habitat type, altitude, active camera trap days of 13 camera trap stations and capture events of red deer, wild boar and wolves at each station

## Additional Information and Declarations

### Field Study Permissions

The following information was supplied relating to field study approvals (i.e., approving body and any reference numbers): Wildlife Department of Turkish Ministry of Agriculture and Forestry approved this study (30057506-030-1867).

### Data Availability

The following information was supplied regarding data availability: The raw measurements are available in the Supplemental Materials.
