# Supplementary material for: Diet and wild ungulate preferences of wolves in northwestern Anatolia during winter
Source: PeerJ. 2019 Aug 21;7:e7446. doi: 10.7717/peerj.7446 (PMC6708370; doi:10.7717/peerj.7446)
Supplement: Supplemental Information 1 — Probably more than one goat was depredated as the wolf pack continued to visit the spot and carry goat parts following several days after depredation. Flock owners have also visited the spot several times searching for separated goat/s. [file peerj-07-7446-s001.pdf]

Mengüllüoğlu D, İlaslan E, Emir H, Berger A. 2019. Diet and wild ungulate preferences of wolves in northwestern Anatolia during winter. PeerJ 7:e7446
http://doi.org/10.7717/peerj.7446

Supplementary File:

Camera trap picture series showing wolf depredation on Angora goat/s separated from the flock and left behind unnoticed by the shepherd in NM. Probably more than one goat was depredated as the wolf pack continued to visit the spot and carry goat parts following several days after depredation. Flock owners have also visited the spot several times searching for separated goat/s.

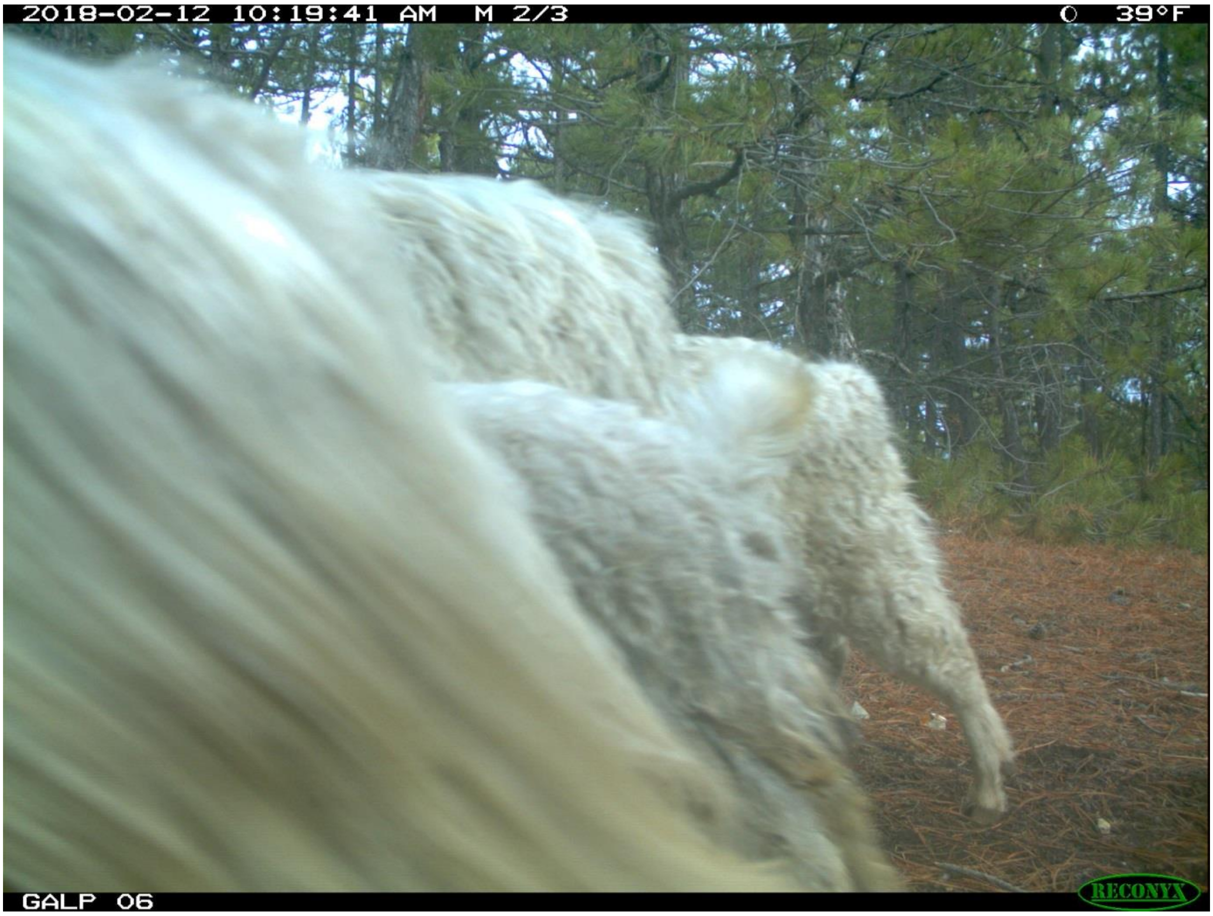

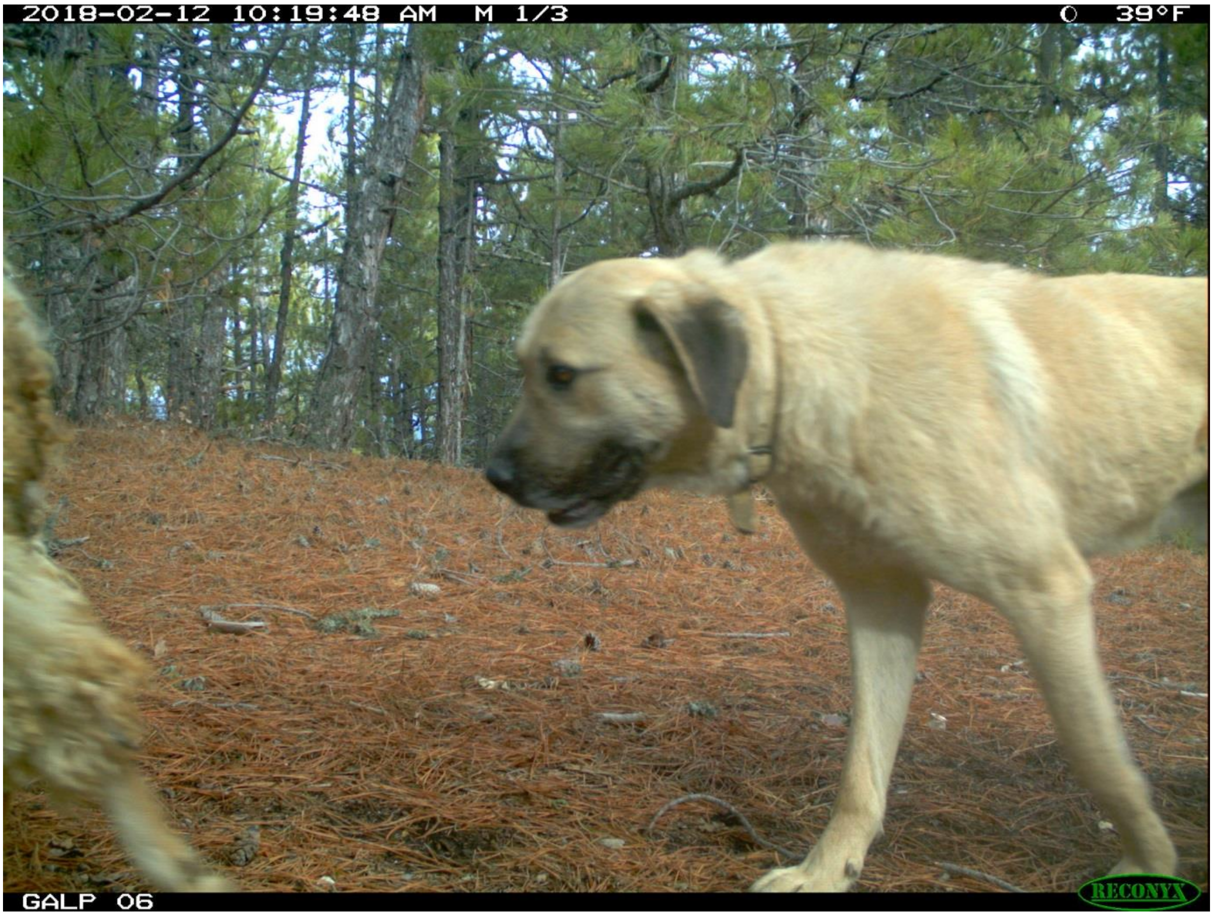
2018-02-12 10:19:48 AM M 1/3
39°F
GALP 06
RECONYX

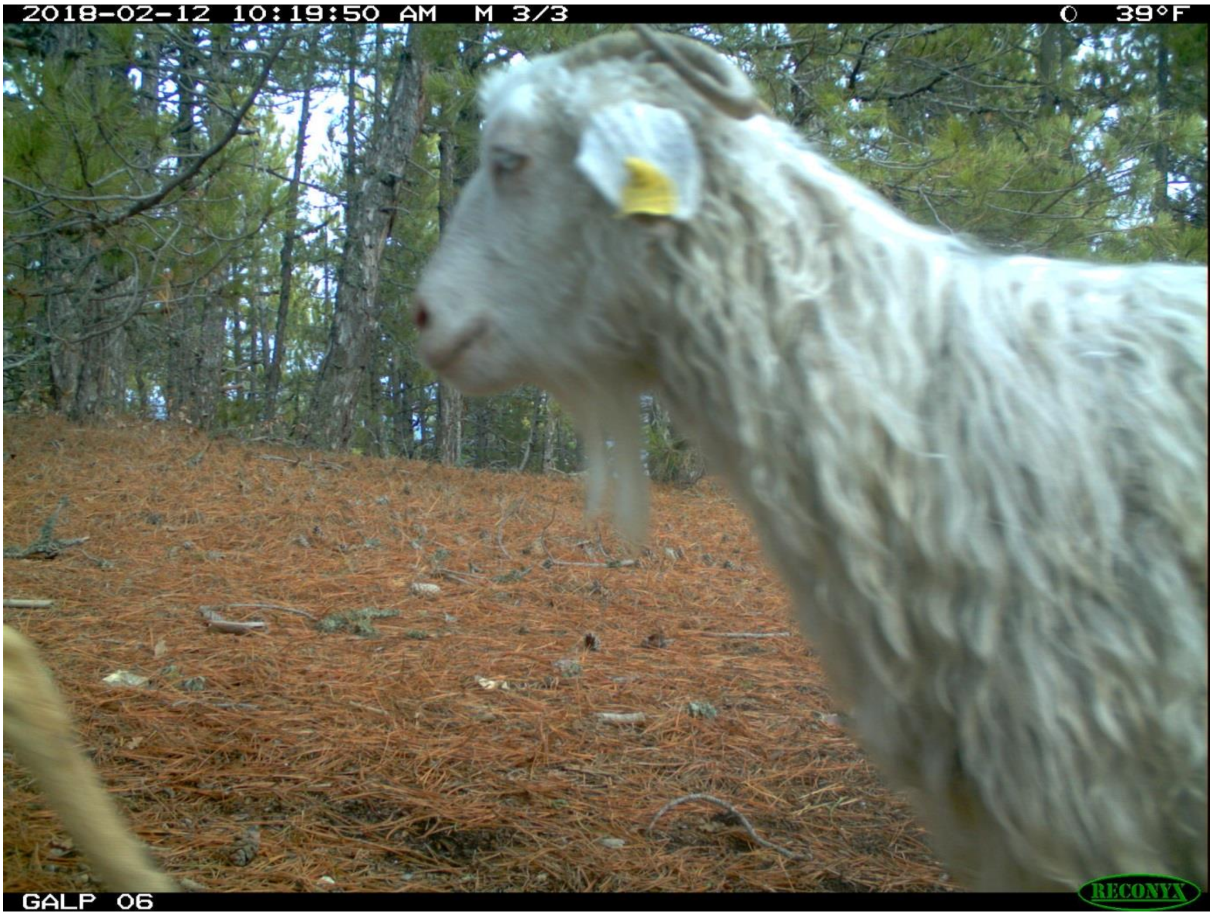
2018-02-12 10:19:50 AM M 3/3
39°F
GALP 06
RECONYX

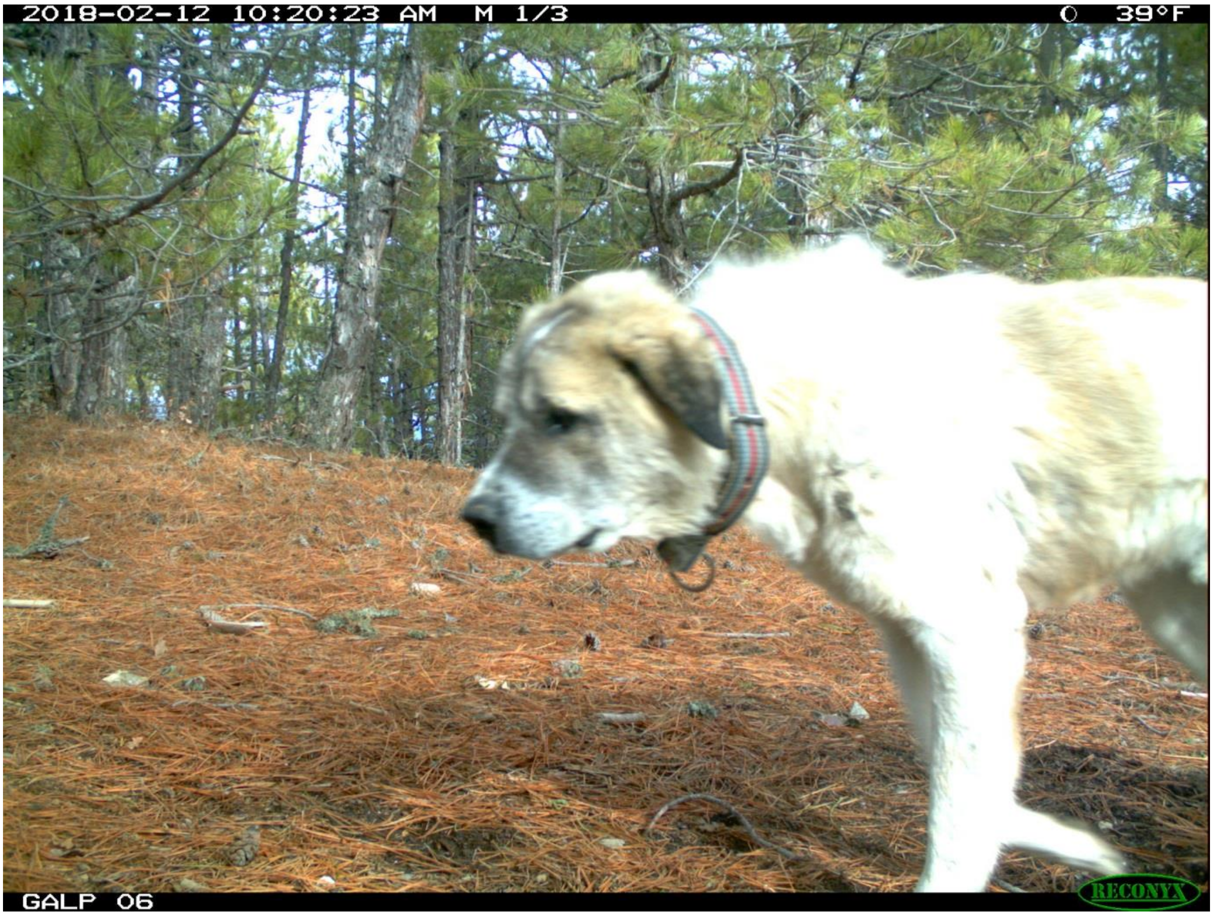
2018-02-12 10:20:23 AM M 1/3
39°F
GALP 06
RECONYX

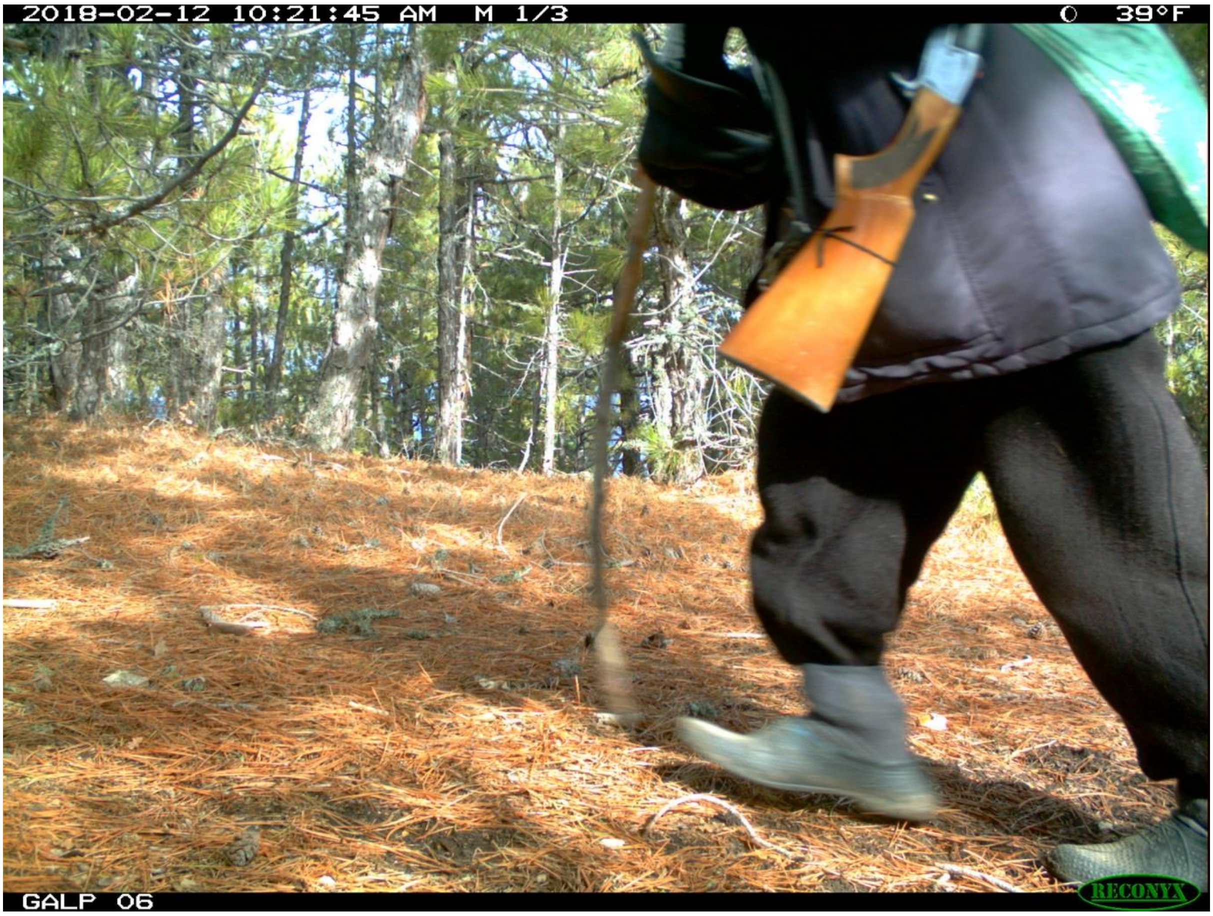
2018-02-12 10:21:45 AM M 1/3
39°F
GALP 06
RECONYX

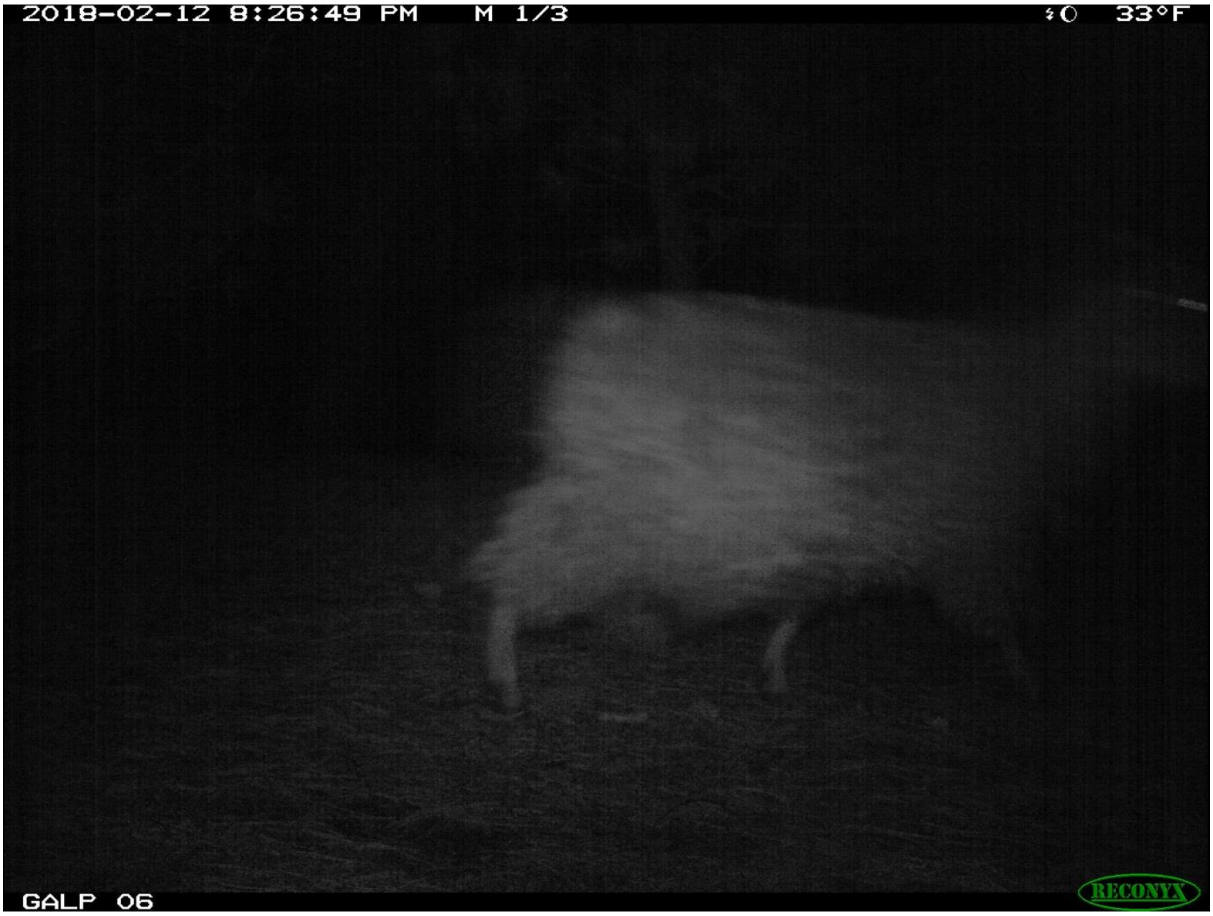
2018-02-12 8:26:49 PM M 1/3 33°F
GALP 06
RECONYX

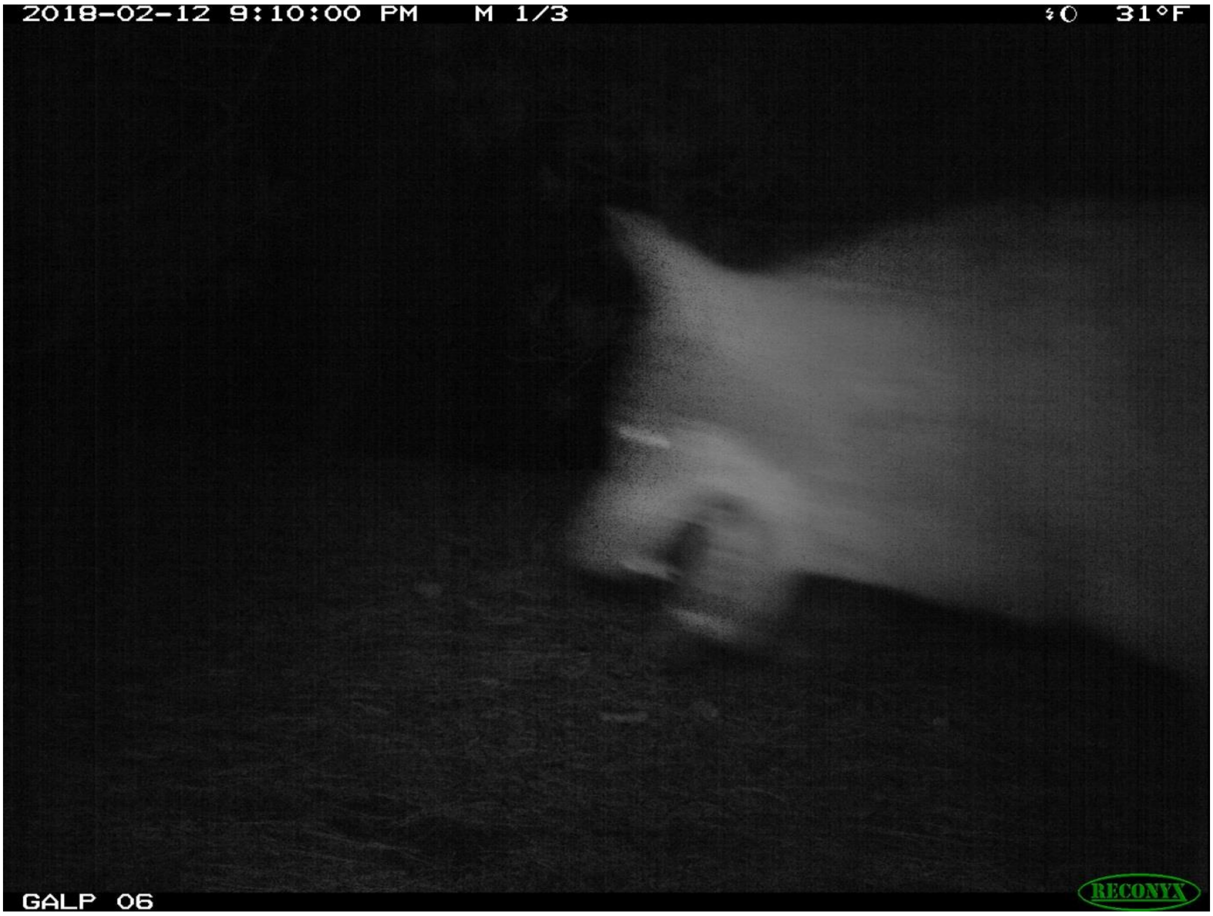
2018-02-12 9:10:00 PM M 1/3 31°F
GALP 06
RECONYX

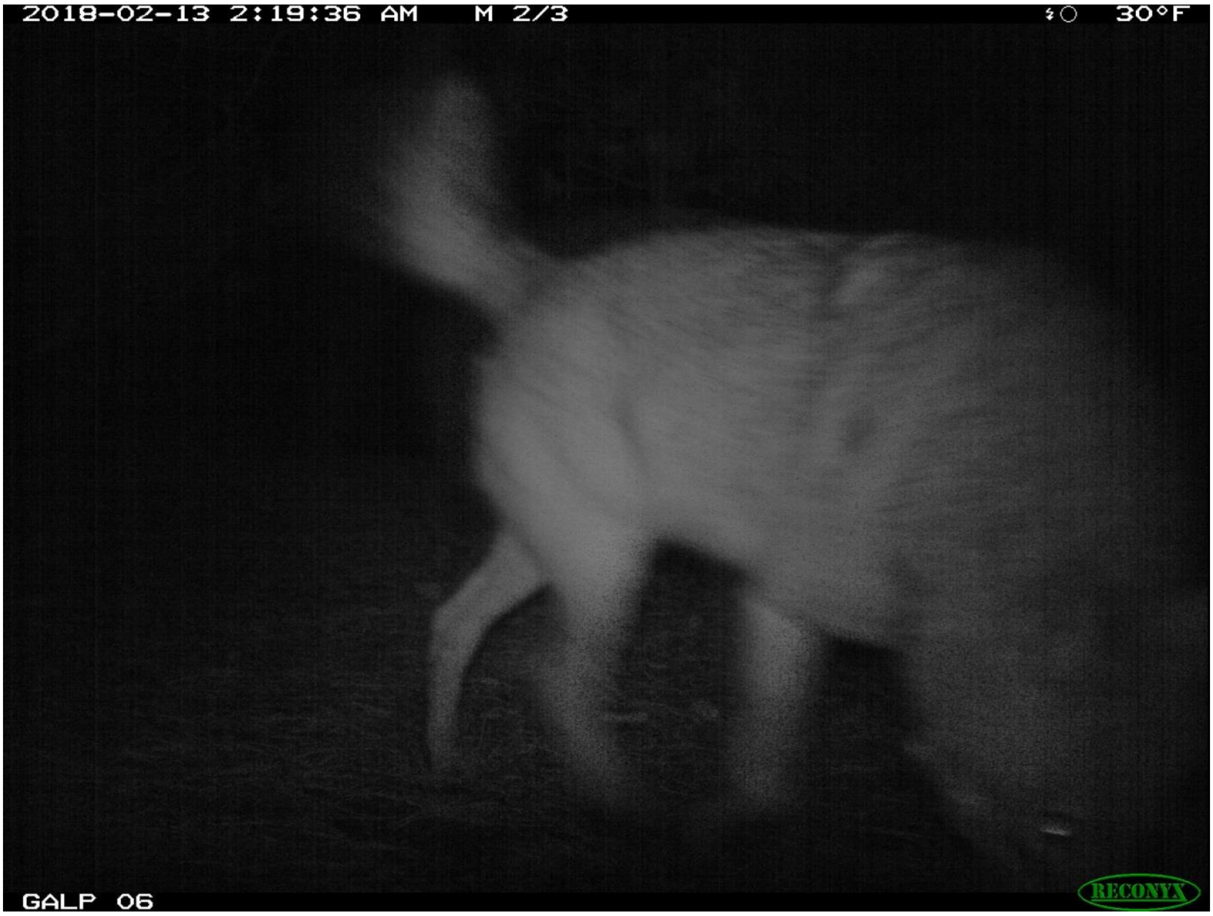
2018-02-13 2:19:36 AM M 2/3
30°F
GALP 06
RECONYX

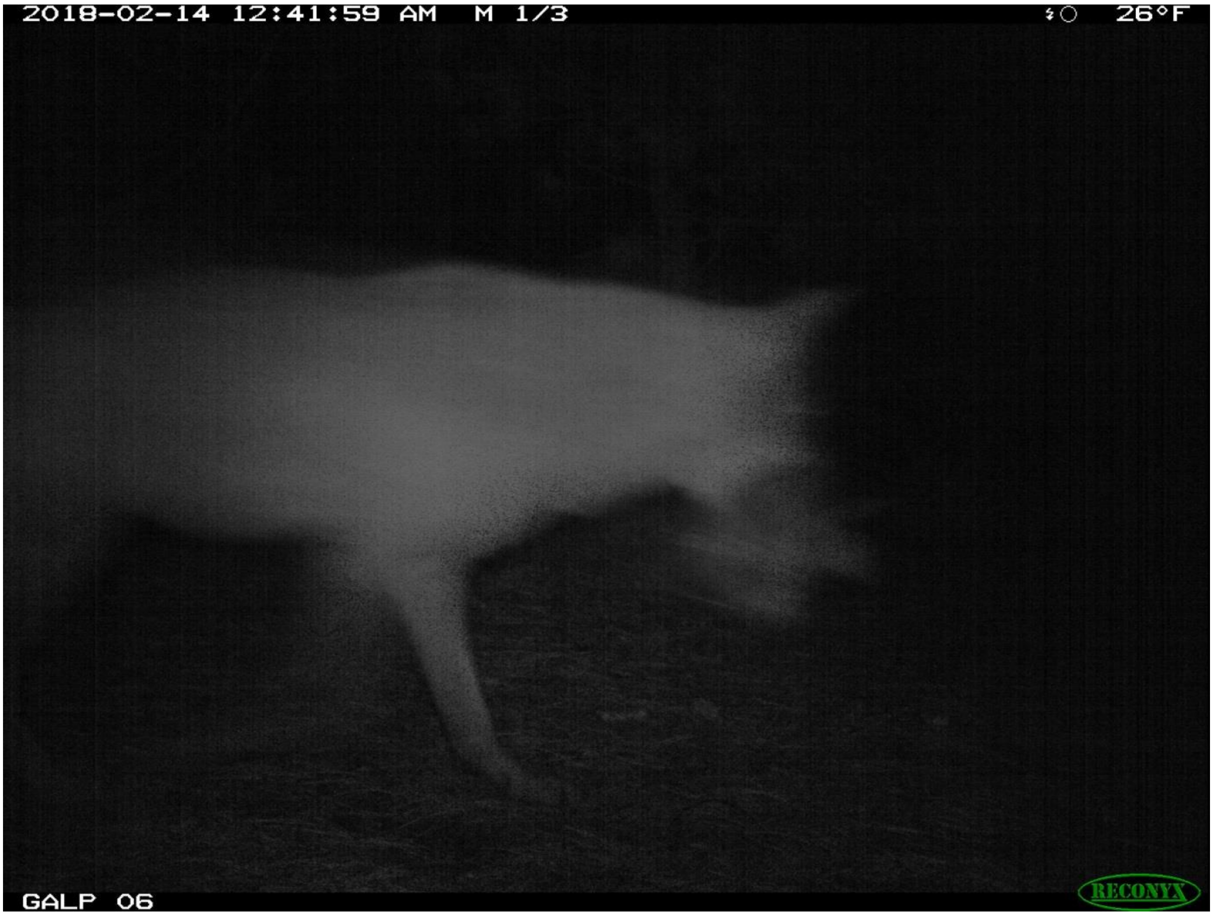
2018-02-14 12:41:59 AM M 1/3
26°F
GALP 06
RECONYX

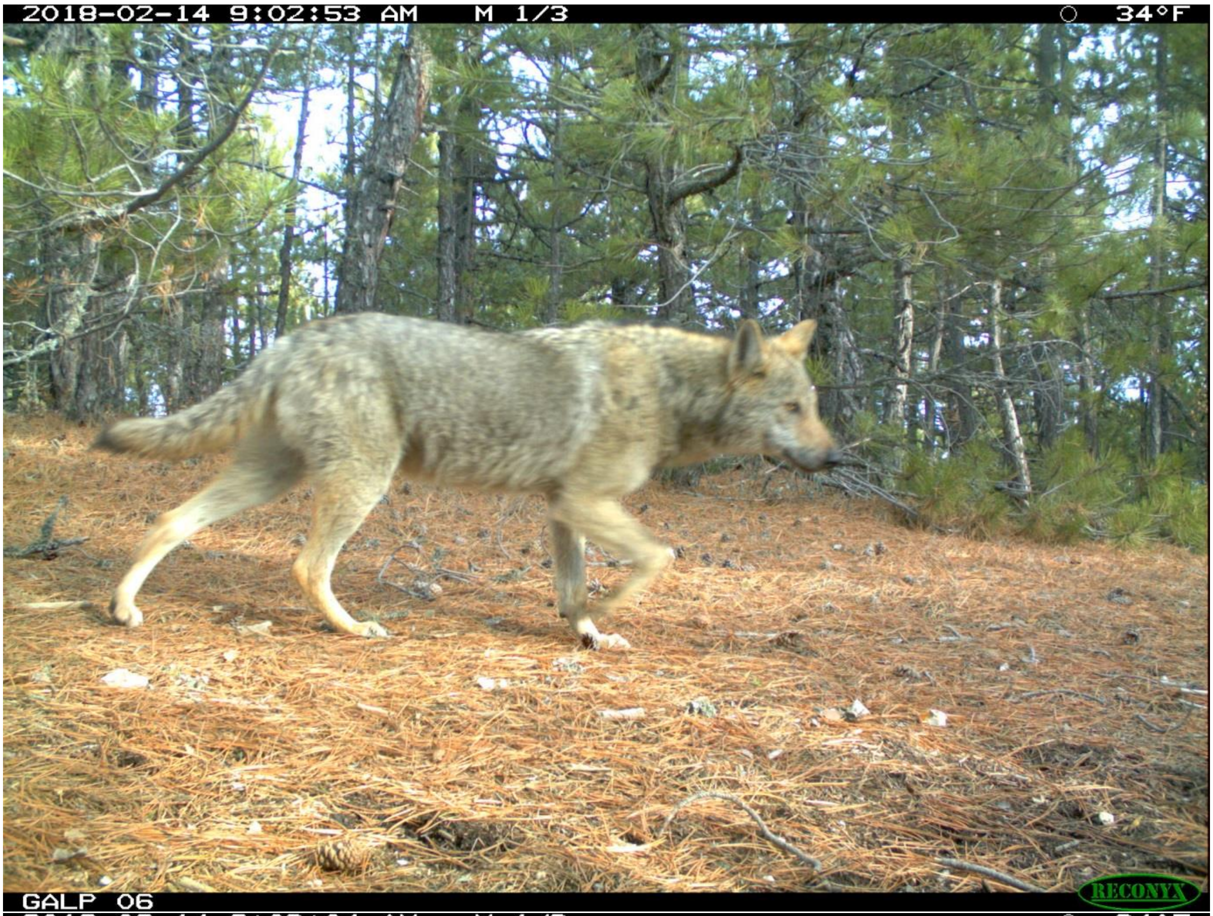
2018-02-14 9:02:53 AM M 1/3
34°F
GALP 06
RECONYX
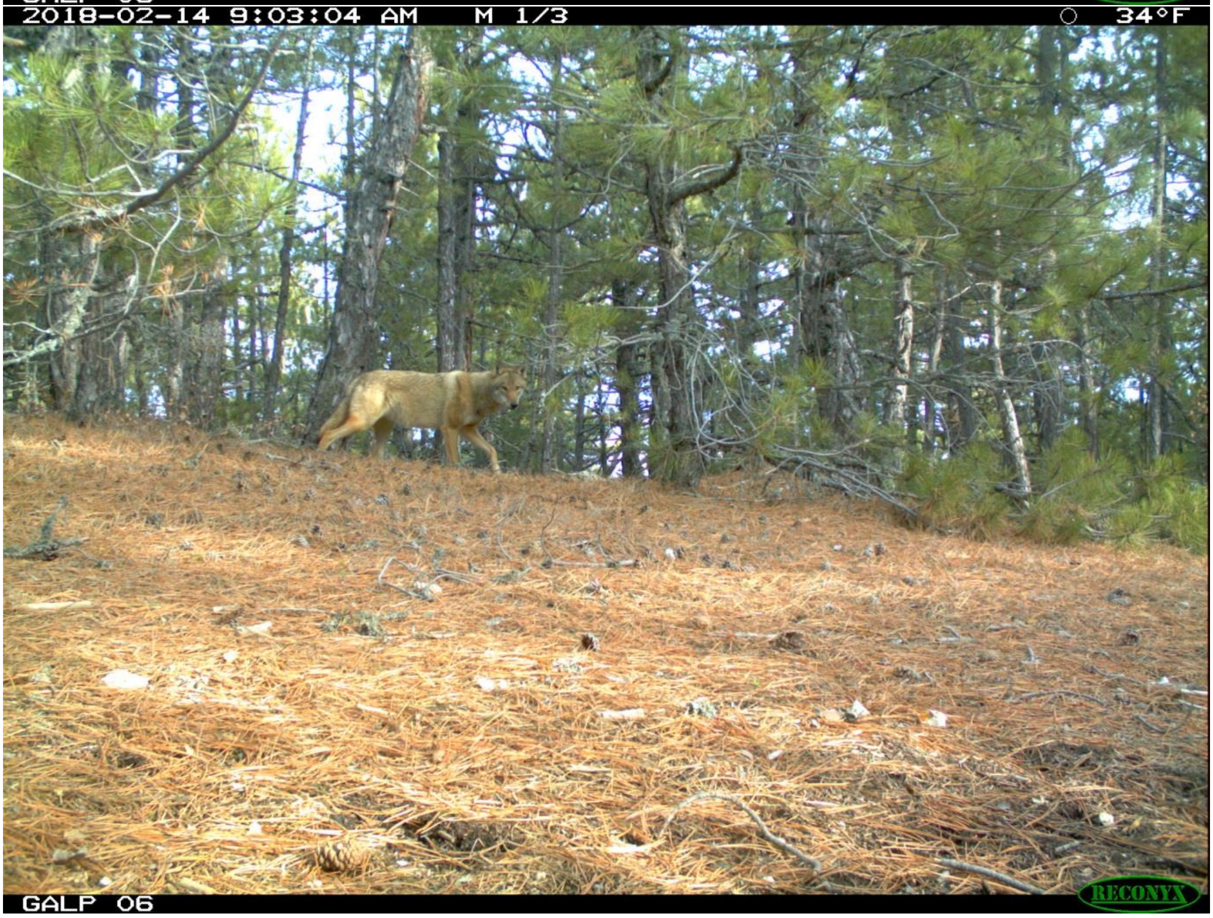
2018-02-14 9:03:04 AM M 1/3
34°F
GALP 06
RECONYX

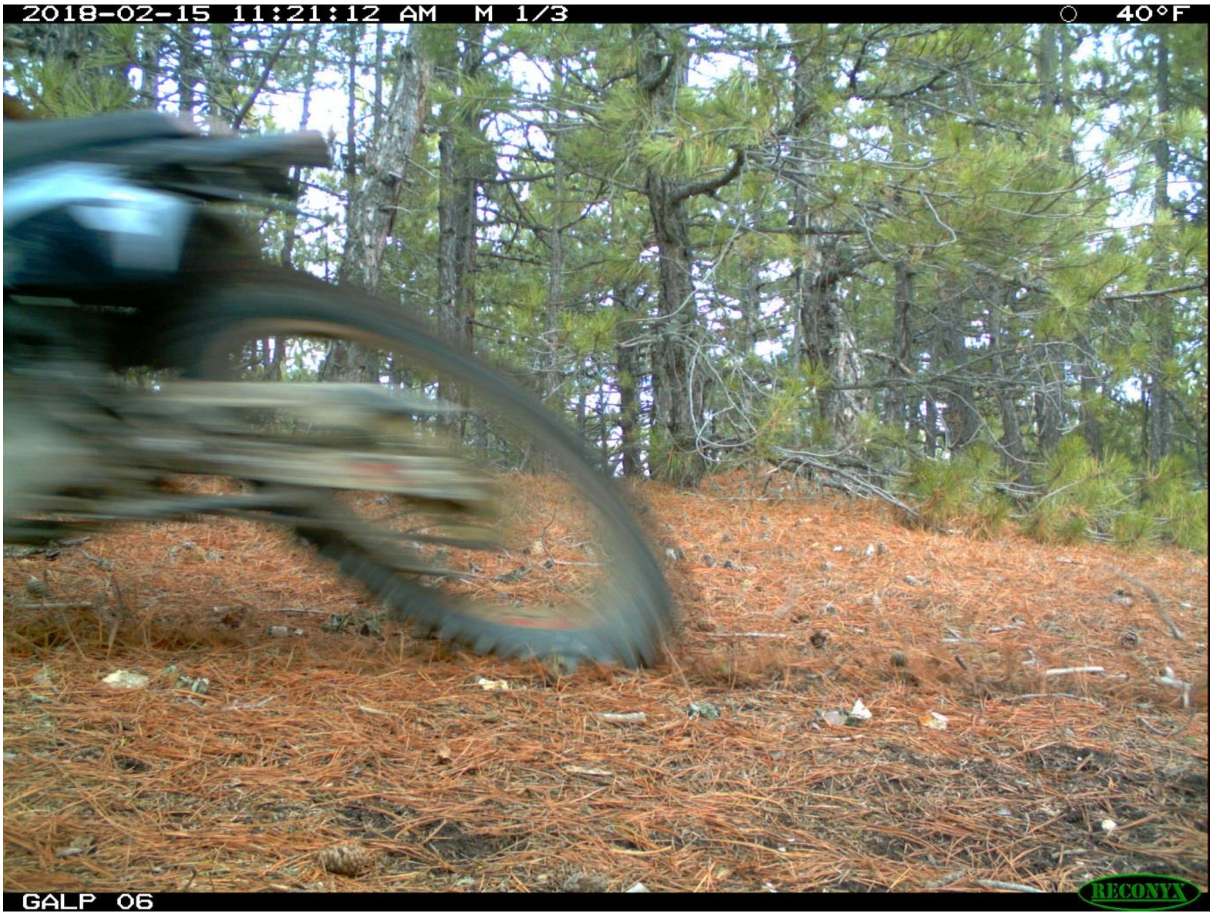
2018-02-15 11:21:12 AM M 1/3
40°F
GALP 06
RECONYX

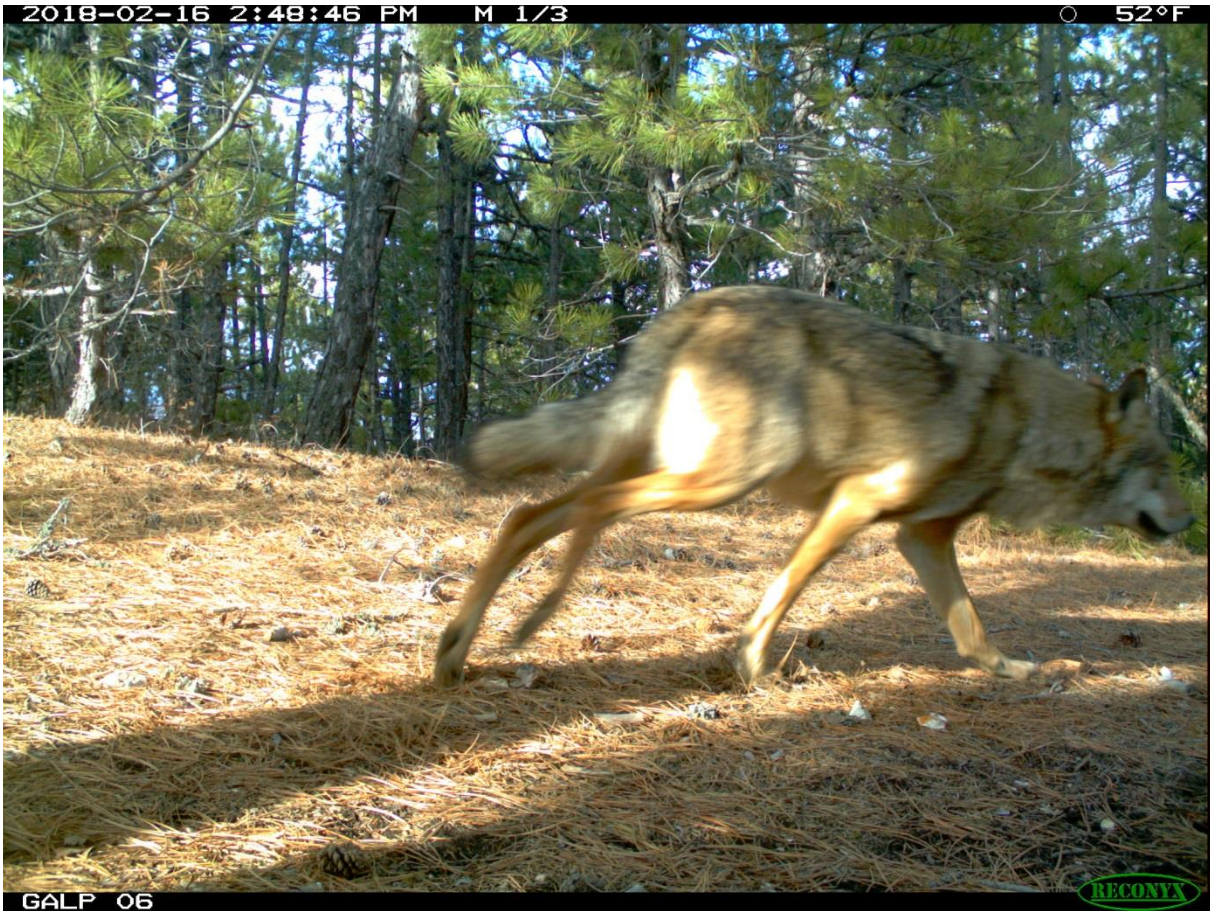
2018-02-16 2:48:46 PM M 1/3
52°F
GALP 06
RECONYX

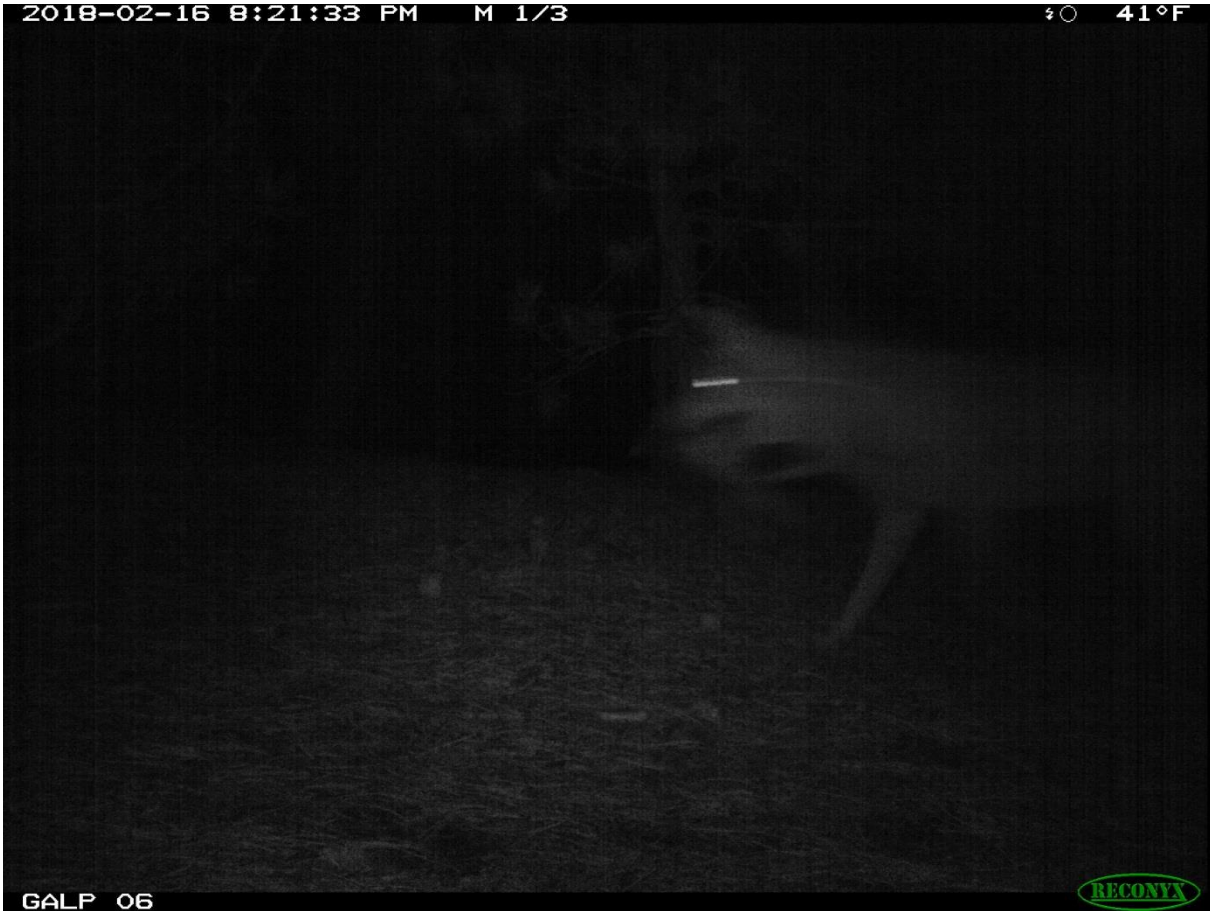
2018-02-16 8:21:33 PM M 1/3
41°F
GALP 06
RECONYX

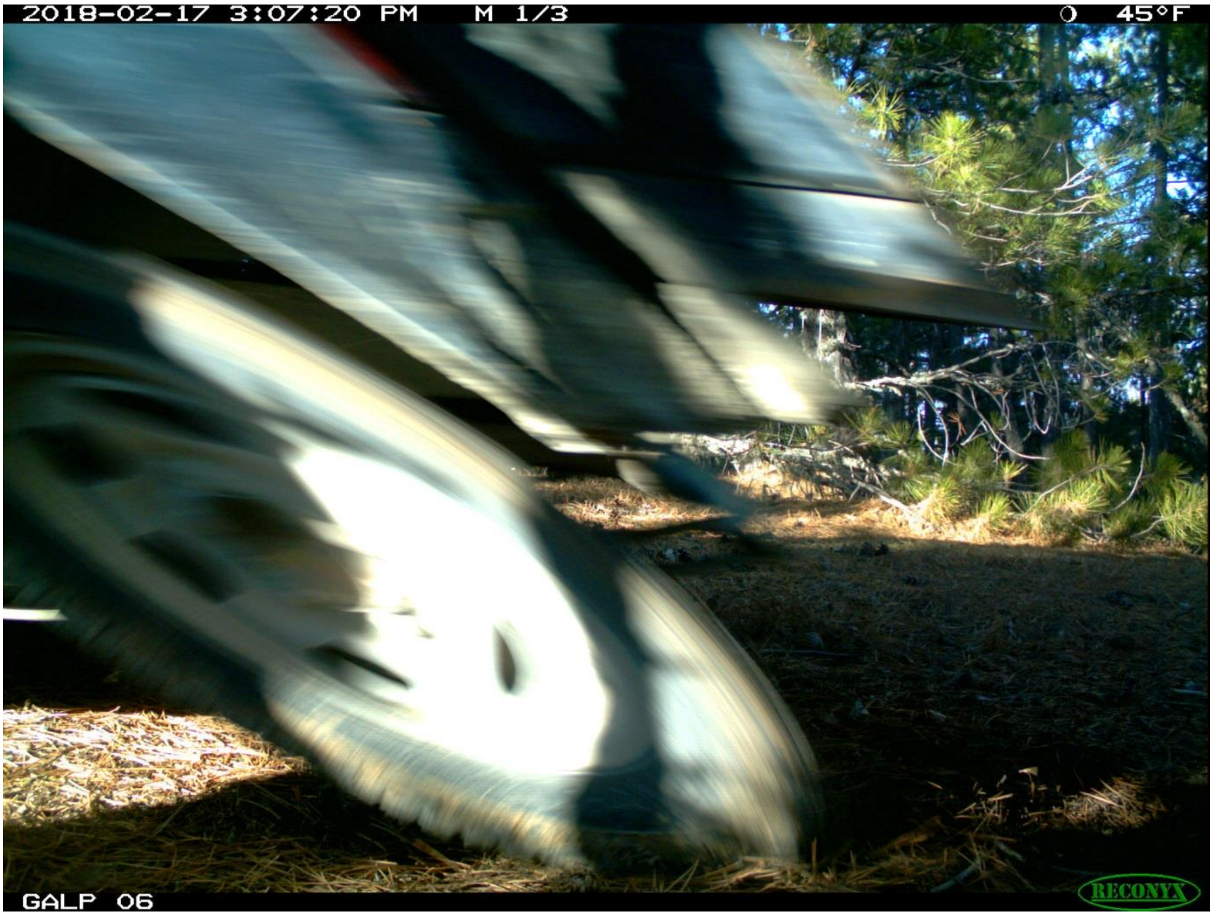
2018-02-17 3:07:20 PM M 1/3
45°F
GALP 06
RECONYX
